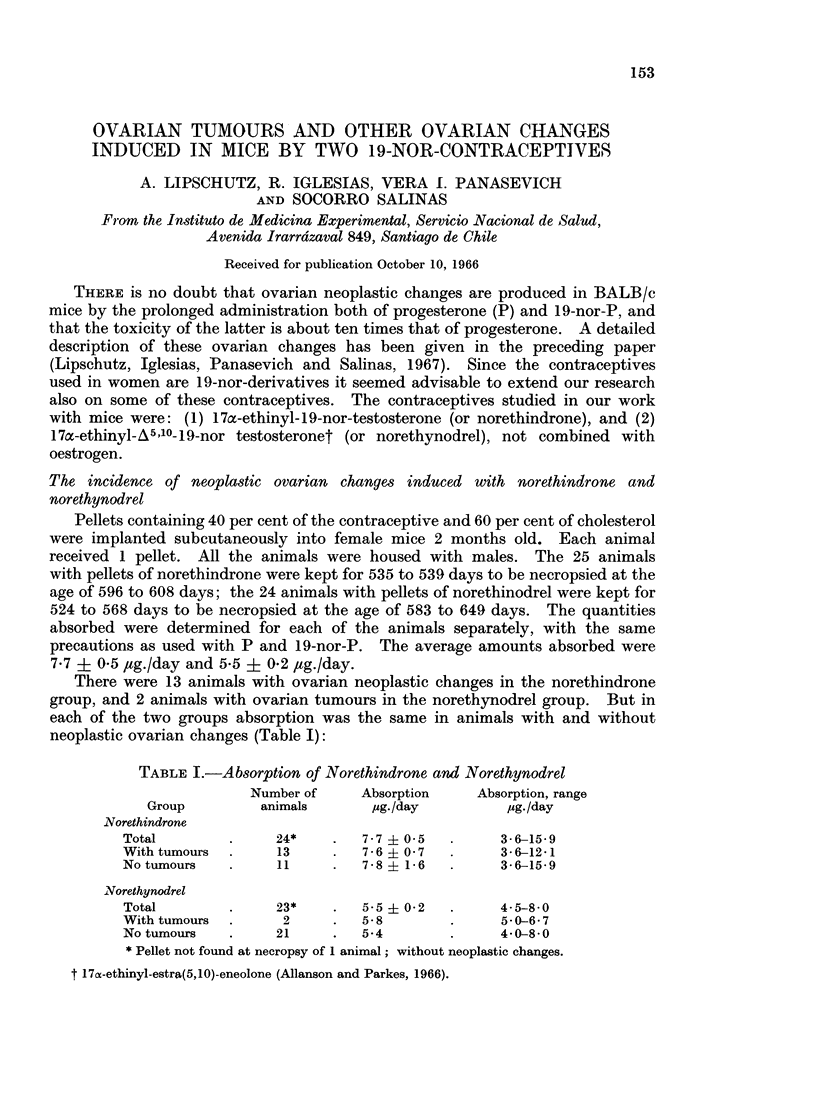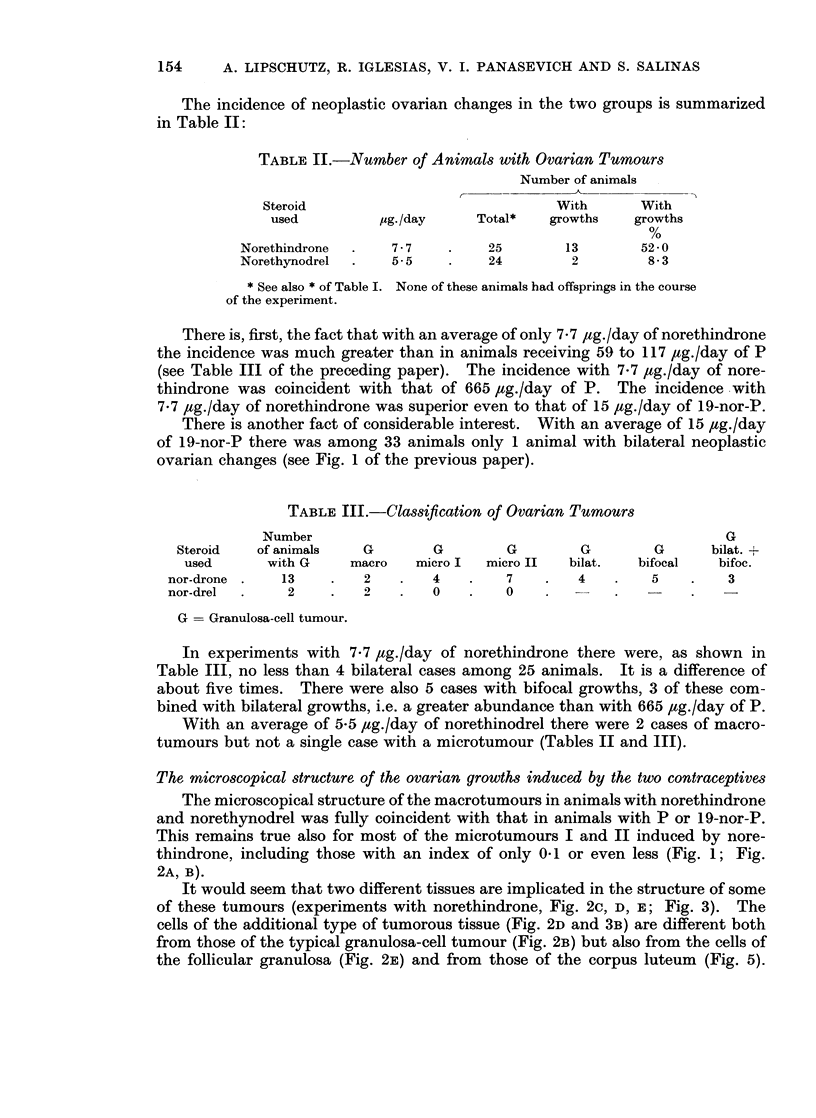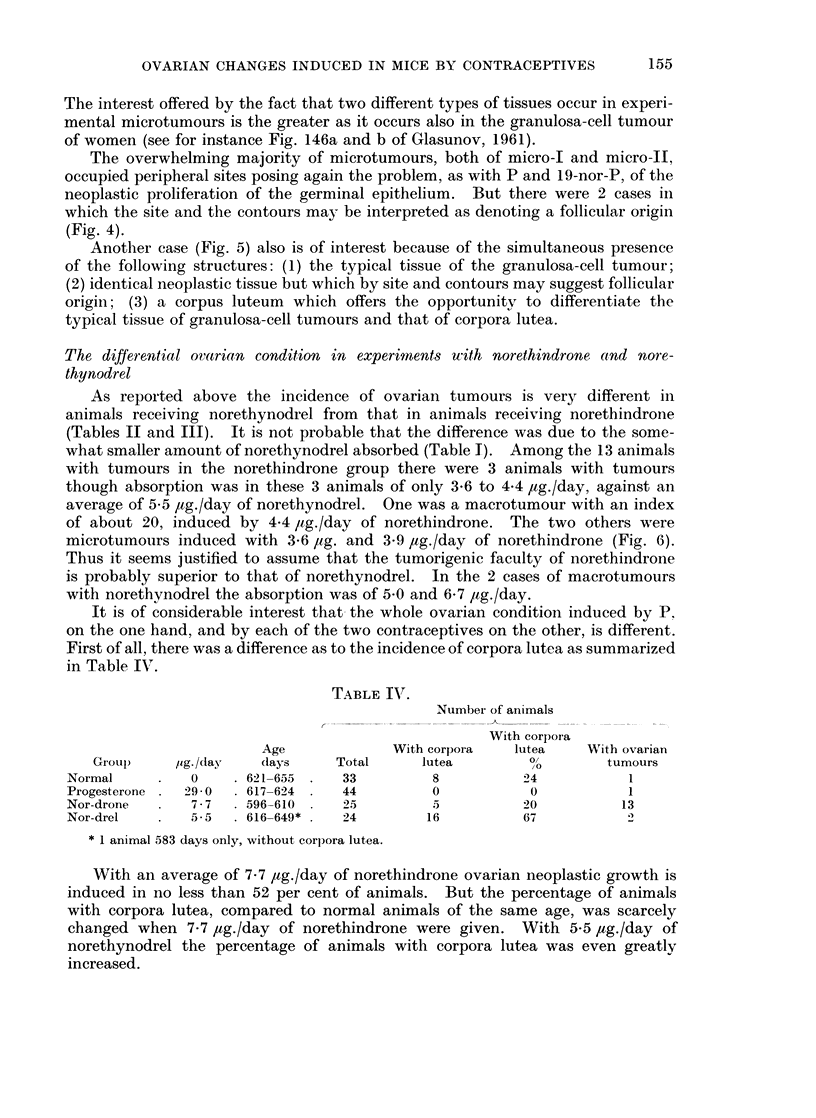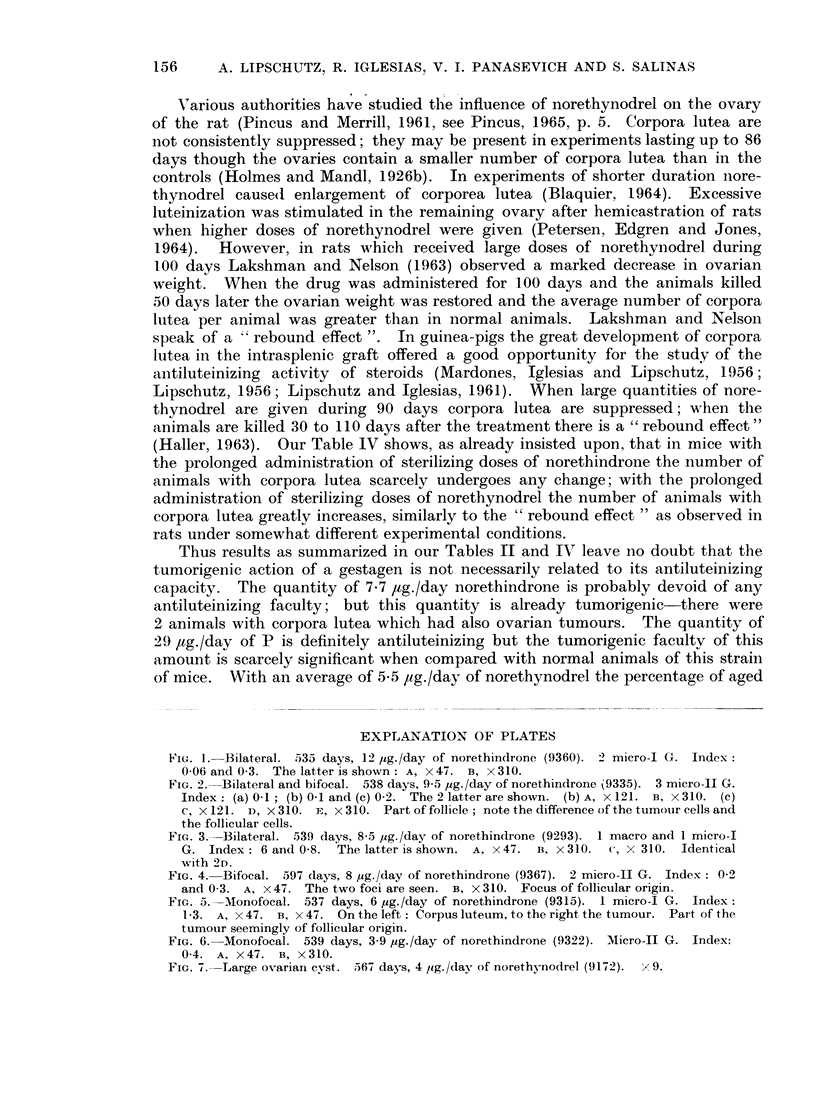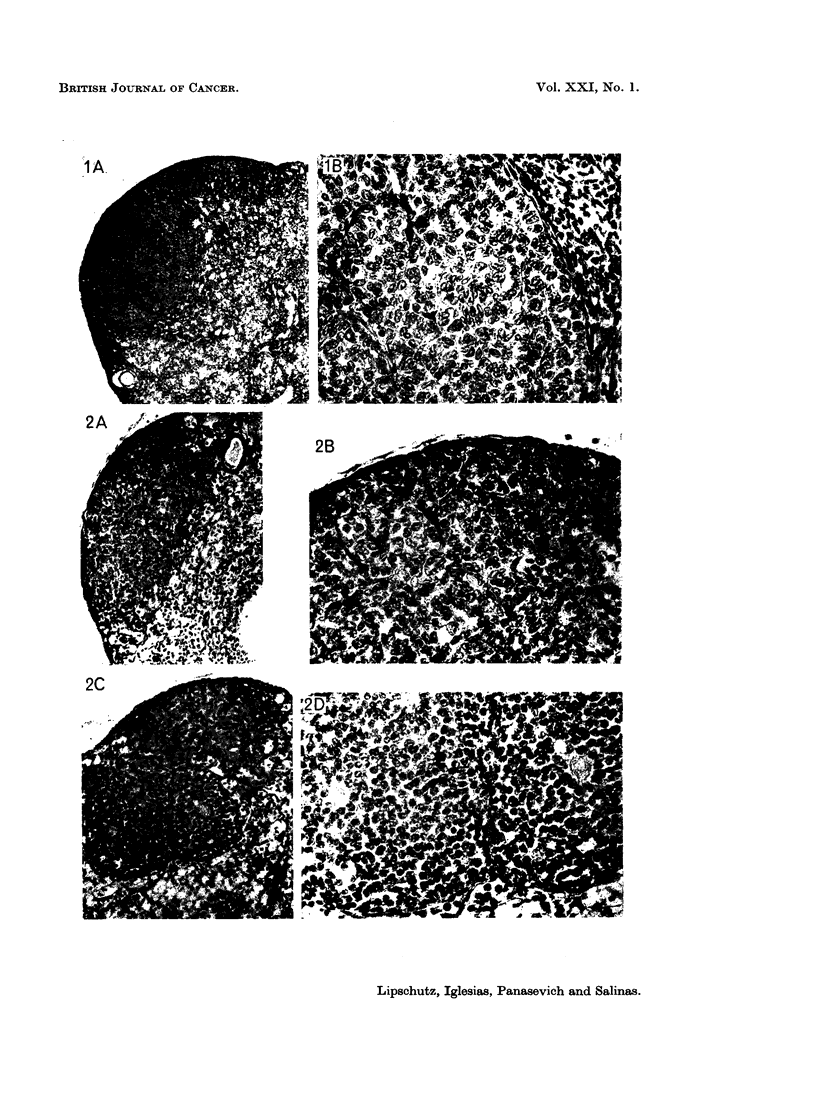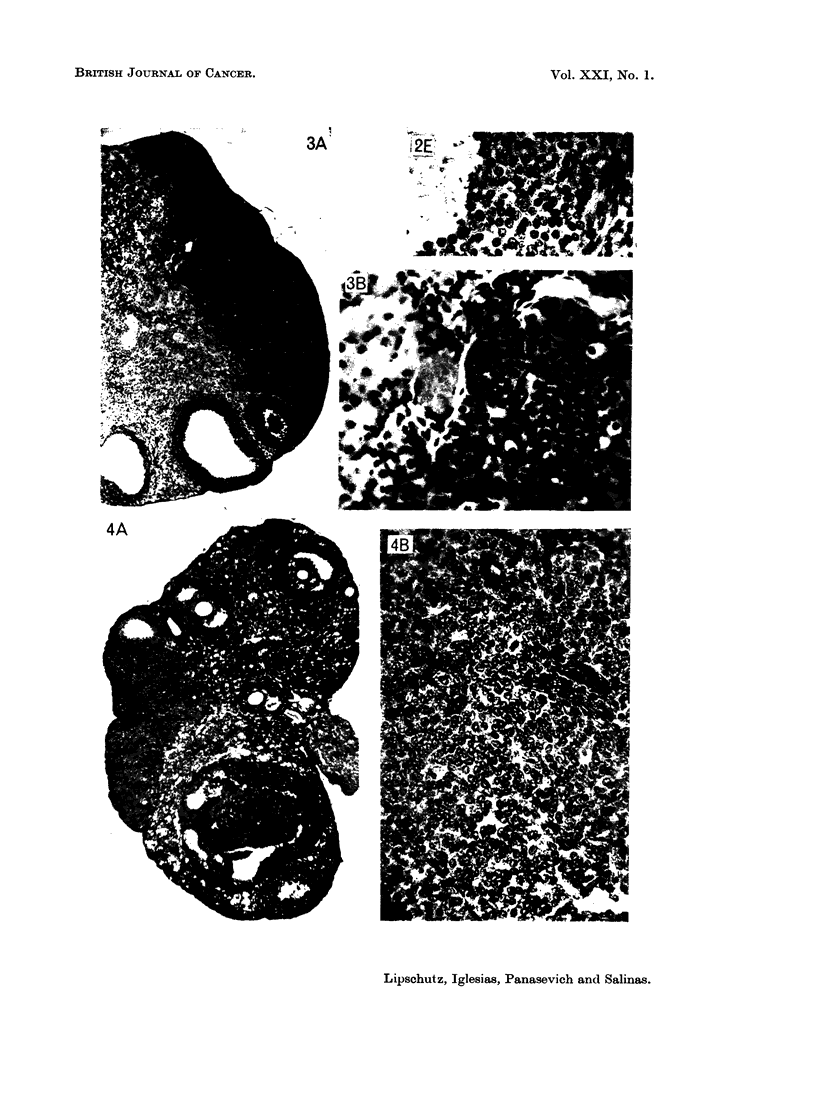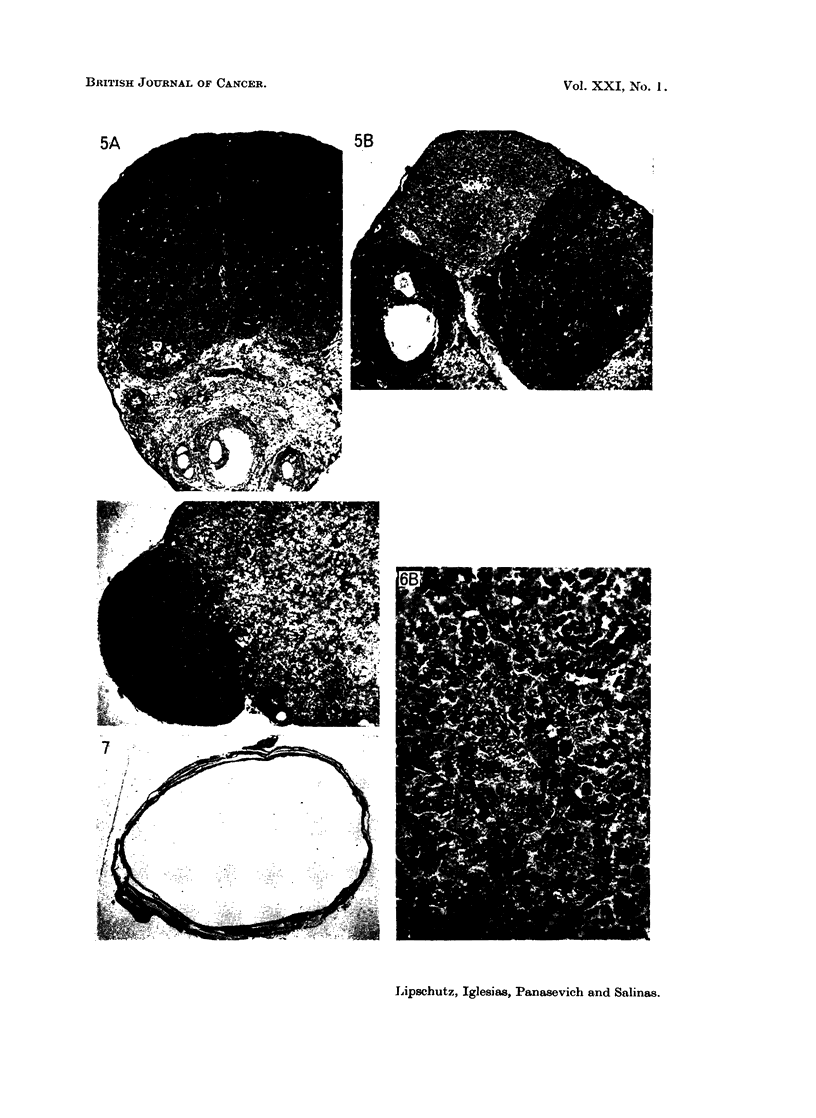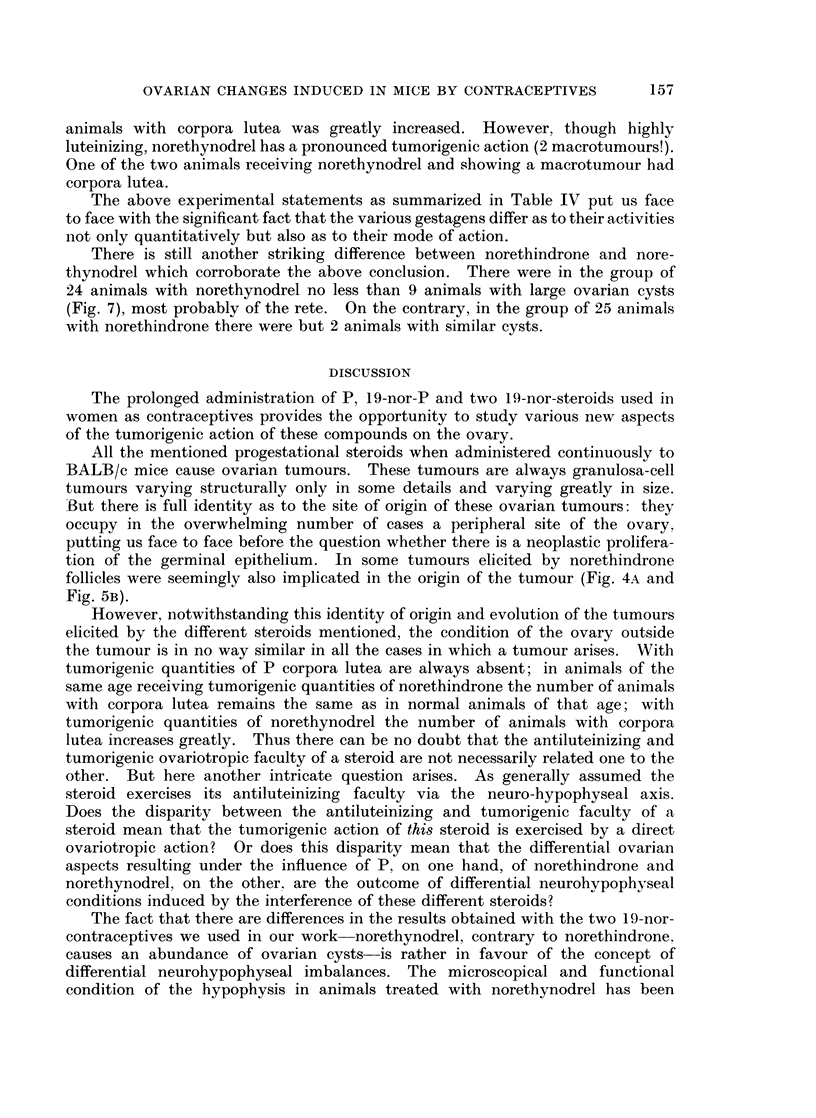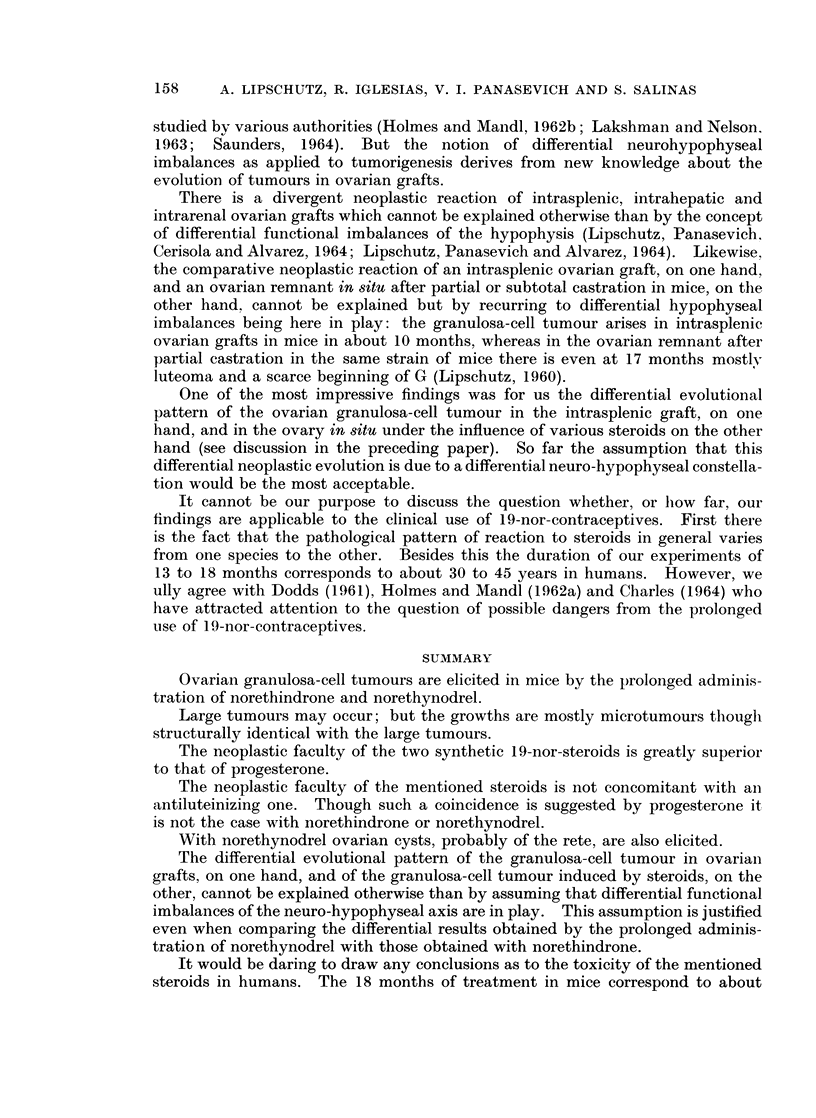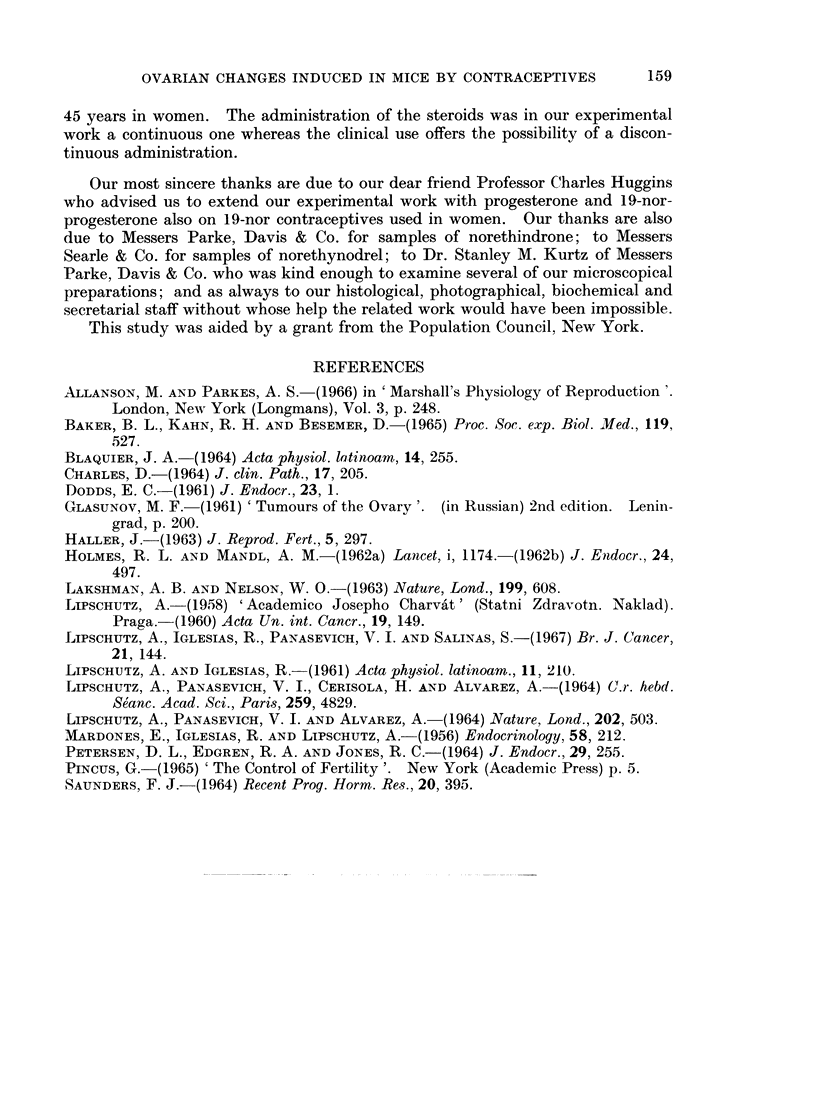# Ovarian tumours and other ovarian changes induced in mice by two 19-nor-contraceptives.

**DOI:** 10.1038/bjc.1967.14

**Published:** 1967-03

**Authors:** A. Lipschutz, R. Iglesias, V. I. Panasevich, S. Salinas

## Abstract

**Images:**


### OVARIAN TUMOURS AND OTHER OVARIAN CHANGES INDUCED IN MICE BY TWO 19-NOR-CONTRACEPTIVES

A. LIPSCHUTZ, R. IGLESIAS, VERA I. PANASEVICH AND SOCORRO SALINAS

*From the Instituto de Medicina Experimental, Servicio Nacional de Salud, Avenida Irarrázaval 849, Santiago de Chile*

Received for publication October 10, 1966

THERE is no doubt that ovarian neoplastic changes are produced in BALB/c mice by the prolonged administration both of progesterone (P) and 19-nor-P, and that the toxicity of the latter is about ten times that of progesterone. A detailed description of these ovarian changes has been given in the preceding paper (Lipschutz, Iglesias, Panasevich and Salinas, 1967). Since the contraceptives used in women are 19-nor-derivatives it seemed advisable to extend our research also on some of these contraceptives. The contraceptives studied in our work with mice were: (1) 17α-ethinyl-19-nor-testosterone (or norethindrone), and (2) 17α-ethinyl-$\Delta^{5,10}$-19-nor testosterone† (or norethynodrel), not combined with oestrogen.

*The incidence of neoplastic ovarian changes induced with norethindrone and norethynodrel*

Pellets containing 40 per cent of the contraceptive and 60 per cent of cholesterol were implanted subcutaneously into female mice 2 months old. Each animal received 1 pellet. All the animals were housed with males. The 25 animals with pellets of norethindrone were kept for 535 to 539 days to be necropsied at the age of 596 to 608 days; the 24 animals with pellets of norethinodrel were kept for 524 to 568 days to be necropsied at the age of 583 to 649 days. The quantities absorbed were determined for each of the animals separately, with the same precautions as used with P and 19-nor-P. The average amounts absorbed were 7·7 ± 0·5 μg./day and 5·5 ± 0·2 μg./day.

There were 13 animals with ovarian neoplastic changes in the norethindrone group, and 2 animals with ovarian tumours in the norethynodrel group. But in each of the two groups absorption was the same in animals with and without neoplastic ovarian changes (Table I):

TABLE I.—*Absorption of Norethindrone and Norethynodrel*

| Group | Number of animals | Absorption μg./day | Absorption, range μg./day |
|---|---|---|---|
| *Norethindrone* | | | |
| Total | 24* | 7·7 ± 0·5 | 3·6–15·9 |
| With tumours | 13 | 7·6 ± 0·7 | 3·6–12·1 |
| No tumours | 11 | 7·8 ± 1·6 | 3·6–15·9 |
| *Norethynodrel* | | | |
| Total | 23* | 5·5 ± 0·2 | 4·5–8·0 |
| With tumours | 2 | 5·8 | 5·0–6·7 |
| No tumours | 21 | 5·4 | 4·0–8·0 |

* Pellet not found at necropsy of 1 animal ; without neoplastic changes.

† 17α-ethinyl-estra(5,10)-eneolone (Allanson and Parkes, 1966).

The incidence of neoplastic ovarian changes in the two groups is summarized in Table II:

TABLE II.—*Number of Animals with Ovarian Tumours*

| Steroid used | μg./day | Number of animals: Total* | With growths | With growths % |
|---|---|---|---|---|
| Norethindrone | 7·7 | 25 | 13 | 52·0 |
| Norethynodrel | 5·5 | 24 | 2 | 8·3 |

* See also * of Table I. None of these animals had offsprings in the course of the experiment.

There is, first, the fact that with an average of only 7·7 μg./day of norethindrone the incidence was much greater than in animals receiving 59 to 117 μg./day of P (see Table III of the preceding paper). The incidence with 7·7 μg./day of norethindrone was coincident with that of 665 μg./day of P. The incidence with 7·7 μg./day of norethindrone was superior even to that of 15 μg./day of 19-nor-P.

There is another fact of considerable interest. With an average of 15 μg./day of 19-nor-P there was among 33 animals only 1 animal with bilateral neoplastic ovarian changes (see Fig. 1 of the previous paper).

TABLE III.—*Classification of Ovarian Tumours*

| Steroid used | Number of animals with G | G macro | G micro I | G micro II | G bilat. | G bifocal | G bilat. + bifoc. |
|---|---|---|---|---|---|---|---|
| nor-drone | 13 | 2 | 4 | 7 | 4 | 5 | 3 |
| nor-drel | 2 | 2 | 0 | 0 | — | — | — |

G = Granulosa-cell tumour.

In experiments with 7·7 μg./day of norethindrone there were, as shown in Table III, no less than 4 bilateral cases among 25 animals. It is a difference of about five times. There were also 5 cases with bifocal growths, 3 of these combined with bilateral growths, i.e. a greater abundance than with 665 μg./day of P.

With an average of 5·5 μg./day of norethinodrel there were 2 cases of macrotumours but not a single case with a microtumour (Tables II and III).

*The microscopical structure of the ovarian growths induced by the two contraceptives*

The microscopical structure of the macrotumours in animals with norethindrone and norethynodrel was fully coincident with that in animals with P or 19-nor-P. This remains true also for most of the microtumours I and II induced by norethindrone, including those with an index of only 0·1 or even less (Fig. 1; Fig. 2A, B).

It would seem that two different tissues are implicated in the structure of some of these tumours (experiments with norethindrone, Fig. 2C, D, E; Fig. 3). The cells of the additional type of tumorous tissue (Fig. 2D and 3B) are different both from those of the typical granulosa-cell tumour (Fig. 2B) but also from the cells of the follicular granulosa (Fig. 2E) and from those of the corpus luteum (Fig. 5).

The interest offered by the fact that two different types of tissues occur in experimental microtumours is the greater as it occurs also in the granulosa-cell tumour of women (see for instance Fig. 146a and b of Glasunov, 1961).

The overwhelming majority of microtumours, both of micro-I and micro-II, occupied peripheral sites posing again the problem, as with P and 19-nor-P, of the neoplastic proliferation of the germinal epithelium. But there were 2 cases in which the site and the contours may be interpreted as denoting a follicular origin (Fig. 4).

Another case (Fig. 5) also is of interest because of the simultaneous presence of the following structures: (1) the typical tissue of the granulosa-cell tumour; (2) identical neoplastic tissue but which by site and contours may suggest follicular origin; (3) a corpus luteum which offers the opportunity to differentiate the typical tissue of granulosa-cell tumours and that of corpora lutea.

*The differential ovarian condition in experiments with norethindrone and norethynodrel*

As reported above the incidence of ovarian tumours is very different in animals receiving norethynodrel from that in animals receiving norethindrone (Tables II and III). It is not probable that the difference was due to the somewhat smaller amount of norethynodrel absorbed (Table I). Among the 13 animals with tumours in the norethindrone group there were 3 animals with tumours though absorption was in these 3 animals of only 3·6 to 4·4 μg./day, against an average of 5·5 μg./day of norethynodrel. One was a macrotumour with an index of about 20, induced by 4·4 μg./day of norethindrone. The two others were microtumours induced with 3·6 μg. and 3·9 μg./day of norethindrone (Fig. 6). Thus it seems justified to assume that the tumorigenic faculty of norethindrone is probably superior to that of norethynodrel. In the 2 cases of macrotumours with norethynodrel the absorption was of 5·0 and 6·7 μg./day.

It is of considerable interest that the whole ovarian condition induced by P, on the one hand, and by each of the two contraceptives on the other, is different. First of all, there was a difference as to the incidence of corpora lutea as summarized in Table IV.

TABLE IV.

| | | | Number of animals | | | |
|---|---|---|---|---|---|---|
| Group | μg./day | Age days | Total | With corpora lutea | With corpora lutea % | With ovarian tumours |
| Normal | 0 | 621–655 | 33 | 8 | 24 | 1 |
| Progesterone | 29·0 | 617–624 | 44 | 0 | 0 | 1 |
| Nor-drone | 7·7 | 596–610 | 25 | 5 | 20 | 13 |
| Nor-drel | 5·5 | 616–649* | 24 | 16 | 67 | 2 |

* 1 animal 583 days only, without corpora lutea.

With an average of 7·7 μg./day of norethindrone ovarian neoplastic growth is induced in no less than 52 per cent of animals. But the percentage of animals with corpora lutea, compared to normal animals of the same age, was scarcely changed when 7·7 μg./day of norethindrone were given. With 5·5 μg./day of norethynodrel the percentage of animals with corpora lutea was even greatly increased.

Various authorities have studied the influence of norethynodrel on the ovary of the rat (Pincus and Merrill, 1961, see Pincus, 1965, p. 5. Corpora lutea are not consistently suppressed; they may be present in experiments lasting up to 86 days though the ovaries contain a smaller number of corpora lutea than in the controls (Holmes and Mandl, 1926b). In experiments of shorter duration norethynodrel caused enlargement of corporea lutea (Blaquier, 1964). Excessive luteinization was stimulated in the remaining ovary after hemicastration of rats when higher doses of norethynodrel were given (Petersen, Edgren and Jones, 1964). However, in rats which received large doses of norethynodrel during 100 days Lakshman and Nelson (1963) observed a marked decrease in ovarian weight. When the drug was administered for 100 days and the animals killed 50 days later the ovarian weight was restored and the average number of corpora lutea per animal was greater than in normal animals. Lakshman and Nelson speak of a "rebound effect". In guinea-pigs the great development of corpora lutea in the intrasplenic graft offered a good opportunity for the study of the antiluteinizing activity of steroids (Mardones, Iglesias and Lipschutz, 1956; Lipschutz, 1956; Lipschutz and Iglesias, 1961). When large quantities of norethynodrel are given during 90 days corpora lutea are suppressed; when the animals are killed 30 to 110 days after the treatment there is a "rebound effect" (Haller, 1963). Our Table IV shows, as already insisted upon, that in mice with the prolonged administration of sterilizing doses of norethindrone the number of animals with corpora lutea scarcely undergoes any change; with the prolonged administration of sterilizing doses of norethynodrel the number of animals with corpora lutea greatly increases, similarly to the "rebound effect" as observed in rats under somewhat different experimental conditions.

Thus results as summarized in our Tables II and IV leave no doubt that the tumorigenic action of a gestagen is not necessarily related to its antiluteinizing capacity. The quantity of 7·7 $\mu$g./day norethindrone is probably devoid of any antiluteinizing faculty; but this quantity is already tumorigenic—there were 2 animals with corpora lutea which had also ovarian tumours. The quantity of 29 $\mu$g./day of P is definitely antiluteinizing but the tumorigenic faculty of this amount is scarcely significant when compared with normal animals of this strain of mice. With an average of 5·5 $\mu$g./day of norethynodrel the percentage of aged

---

EXPLANATION OF PLATES

FIG. 1.—Bilateral. 535 days, 12 $\mu$g./day of norethindrone (9360). 2 micro-I G. Index: 0·06 and 0·3. The latter is shown: A, ×47. B, ×310.

FIG. 2.—Bilateral and bifocal. 538 days, 9·5 $\mu$g./day of norethindrone (9335). 3 micro-II G. Index: (a) 0·1; (b) 0·1 and (c) 0·2. The 2 latter are shown. (b) A, ×121. B, ×310. (c) C, ×121. D, ×310. E, ×310. Part of follicle; note the difference of the tumour cells and the follicular cells.

FIG. 3.—Bilateral. 539 days, 8·5 $\mu$g./day of norethindrone (9293). 1 macro and 1 micro-I G. Index: 6 and 0·8. The latter is shown. A, ×47. B, ×310. C, ×310. Identical with 2D.

FIG. 4.—Bifocal. 597 days, 8 $\mu$g./day of norethindrone (9367). 2 micro-II G. Index: 0·2 and 0·3. A, ×47. The two foci are seen. B, ×310. Focus of follicular origin.

FIG. 5.—Monofocal. 537 days, 6 $\mu$g./day of norethindrone (9315). 1 micro-I G. Index: 1·3. A, ×47. B, ×47. On the left: Corpus luteum, to the right the tumour. Part of the tumour seemingly of follicular origin.

FIG. 6.—Monofocal. 539 days, 3·9 $\mu$g./day of norethindrone (9322). Micro-II G. Index: 0·4. A, ×47. B, ×310.

FIG. 7.—Large ovarian cyst. 567 days, 4 $\mu$g./day of norethynodrel (9172). ×9.

Lipschutz, Iglesias, Panasevich and Salinas.

3A

2E

3B

4A

4B

5A

5B

6A

6B

7

animals with corpora lutea was greatly increased. However, though highly luteinizing, norethynodrel has a pronounced tumorigenic action (2 macrotumours!). One of the two animals receiving norethynodrel and showing a macrotumour had corpora lutea.

The above experimental statements as summarized in Table IV put us face to face with the significant fact that the various gestagens differ as to their activities not only quantitatively but also as to their mode of action.

There is still another striking difference between norethindrone and norethynodrel which corroborate the above conclusion. There were in the group of 24 animals with norethynodrel no less than 9 animals with large ovarian cysts (Fig. 7), most probably of the rete. On the contrary, in the group of 25 animals with norethindrone there were but 2 animals with similar cysts.

### DISCUSSION

The prolonged administration of P, 19-nor-P and two 19-nor-steroids used in women as contraceptives provides the opportunity to study various new aspects of the tumorigenic action of these compounds on the ovary.

All the mentioned progestational steroids when administered continuously to BALB/c mice cause ovarian tumours. These tumours are always granulosa-cell tumours varying structurally only in some details and varying greatly in size. But there is full identity as to the site of origin of these ovarian tumours: they occupy in the overwhelming number of cases a peripheral site of the ovary, putting us face to face before the question whether there is a neoplastic proliferation of the germinal epithelium. In some tumours elicited by norethindrone follicles were seemingly also implicated in the origin of the tumour (Fig. 4A and Fig. 5B).

However, notwithstanding this identity of origin and evolution of the tumours elicited by the different steroids mentioned, the condition of the ovary outside the tumour is in no way similar in all the cases in which a tumour arises. With tumorigenic quantities of P corpora lutea are always absent; in animals of the same age receiving tumorigenic quantities of norethindrone the number of animals with corpora lutea remains the same as in normal animals of that age; with tumorigenic quantities of norethynodrel the number of animals with corpora lutea increases greatly. Thus there can be no doubt that the antiluteinizing and tumorigenic ovariotropic faculty of a steroid are not necessarily related one to the other. But here another intricate question arises. As generally assumed the steroid exercises its antiluteinizing faculty via the neuro-hypophyseal axis. Does the disparity between the antiluteinizing and tumorigenic faculty of a steroid mean that the tumorigenic action of *this* steroid is exercised by a direct ovariotropic action? Or does this disparity mean that the differential ovarian aspects resulting under the influence of P, on one hand, of norethindrone and norethynodrel, on the other, are the outcome of differential neurohypophyseal conditions induced by the interference of these different steroids?

The fact that there are differences in the results obtained with the two 19-nor-contraceptives we used in our work—norethynodrel, contrary to norethindrone, causes an abundance of ovarian cysts—is rather in favour of the concept of differential neurohypophyseal imbalances. The microscopical and functional condition of the hypophysis in animals treated with norethynodrel has been studied by various authorities (Holmes and Mandl, 1962b; Lakshman and Nelson, 1963; Saunders, 1964). But the notion of differential neurohypophyseal imbalances as applied to tumorigenesis derives from new knowledge about the evolution of tumours in ovarian grafts.

There is a divergent neoplastic reaction of intrasplenic, intrahepatic and intrarenal ovarian grafts which cannot be explained otherwise than by the concept of differential functional imbalances of the hypophysis (Lipschutz, Panasevich, Cerisola and Alvarez, 1964; Lipschutz, Panasevich and Alvarez, 1964). Likewise, the comparative neoplastic reaction of an intrasplenic ovarian graft, on one hand, and an ovarian remnant *in situ* after partial or subtotal castration in mice, on the other hand, cannot be explained but by recurring to differential hypophyseal imbalances being here in play: the granulosa-cell tumour arises in intrasplenic ovarian grafts in mice in about 10 months, whereas in the ovarian remnant after partial castration in the same strain of mice there is even at 17 months mostly luteoma and a scarce beginning of G (Lipschutz, 1960).

One of the most impressive findings was for us the differential evolutional pattern of the ovarian granulosa-cell tumour in the intrasplenic graft, on one hand, and in the ovary *in situ* under the influence of various steroids on the other hand (see discussion in the preceding paper). So far the assumption that this differential neoplastic evolution is due to a differential neuro-hypophyseal constellation would be the most acceptable.

It cannot be our purpose to discuss the question whether, or how far, our findings are applicable to the clinical use of 19-nor-contraceptives. First there is the fact that the pathological pattern of reaction to steroids in general varies from one species to the other. Besides this the duration of our experiments of 13 to 18 months corresponds to about 30 to 45 years in humans. However, we ully agree with Dodds (1961), Holmes and Mandl (1962a) and Charles (1964) who have attracted attention to the question of possible dangers from the prolonged use of 19-nor-contraceptives.

### SUMMARY

Ovarian granulosa-cell tumours are elicited in mice by the prolonged administration of norethindrone and norethynodrel.

Large tumours may occur; but the growths are mostly microtumours though structurally identical with the large tumours.

The neoplastic faculty of the two synthetic 19-nor-steroids is greatly superior to that of progesterone.

The neoplastic faculty of the mentioned steroids is not concomitant with an antiluteinizing one. Though such a coincidence is suggested by progesterone it is not the case with norethindrone or norethynodrel.

With norethynodrel ovarian cysts, probably of the rete, are also elicited.

The differential evolutional pattern of the granulosa-cell tumour in ovarian grafts, on one hand, and of the granulosa-cell tumour induced by steroids, on the other, cannot be explained otherwise than by assuming that differential functional imbalances of the neuro-hypophyseal axis are in play. This assumption is justified even when comparing the differential results obtained by the prolonged administration of norethynodrel with those obtained with norethindrone.

It would be daring to draw any conclusions as to the toxicity of the mentioned steroids in humans. The 18 months of treatment in mice correspond to about

45 years in women. The administration of the steroids was in our experimental work a continuous one whereas the clinical use offers the possibility of a discontinuous administration.

Our most sincere thanks are due to our dear friend Professor Charles Huggins who advised us to extend our experimental work with progesterone and 19-nor-progesterone also on 19-nor contraceptives used in women. Our thanks are also due to Messers Parke, Davis & Co. for samples of norethindrone; to Messers Searle & Co. for samples of norethynodrel; to Dr. Stanley M. Kurtz of Messers Parke, Davis & Co. who was kind enough to examine several of our microscopical preparations; and as always to our histological, photographical, biochemical and secretarial staff without whose help the related work would have been impossible.

This study was aided by a grant from the Population Council, New York.

### REFERENCES

ALLANSON, M. AND PARKES, A. S.—(1966) in ' Marshall's Physiology of Reproduction '. London, New York (Longmans), Vol. 3, p. 248.

BAKER, B. L., KAHN, R. H. AND BESEMER, D.—(1965) *Proc. Soc. exp. Biol. Med.*, **119**, 527.

BLAQUIER, J. A.—(1964) *Acta physiol. latinoam*, **14**, 255.

CHARLES, D.—(1964) *J. clin. Path.*, **17**, 205.

DODDS, E. C.—(1961) *J. Endocr.*, **23**, 1.

GLASUNOV, M. F.—(1961) ' Tumours of the Ovary '. (in Russian) 2nd edition. Leningrad, p. 200.

HALLER, J.—(1963) *J. Reprod. Fert.*, **5**, 297.

HOLMES, R. L. AND MANDL, A. M.—(1962a) *Lancet*, i, 1174.—(1962b) *J. Endocr.*, **24**, 497.

LAKSHMAN, A. B. AND NELSON, W. O.—(1963) *Nature, Lond.*, **199**, 608.

LIPSCHUTZ, A.—(1958) 'Academico Josepho Charvát' (Statni Zdravotn. Naklad). Praga.—(1960) *Acta Un. int. Cancr.*, **19**, 149.

LIPSCHUTZ, A., IGLESIAS, R., PANASEVICH, V. I. AND SALINAS, S.—(1967) *Br. J. Cancer*, **21**, 144.

LIPSCHUTZ, A. AND IGLESIAS, R.—(1961) *Acta physiol. latinoam.*, **11**, 210.

LIPSCHUTZ, A., PANASEVICH, V. I., CERISOLA, H. AND ALVAREZ, A.—(1964) *C.r. hebd. Séanc. Acad. Sci., Paris*, **259**, 4829.

LIPSCHUTZ, A., PANASEVICH, V. I. AND ALVAREZ, A.—(1964) *Nature, Lond.*, **202**, 503.

MARDONES, E., IGLESIAS, R. AND LIPSCHUTZ, A.—(1956) *Endocrinology*, **58**, 212.

PETERSEN, D. L., EDGREN, R. A. AND JONES, R. C.—(1964) *J. Endocr.*, **29**, 255.

PINCUS, G.—(1965) ' The Control of Fertility '. New York (Academic Press) p. 5.

SAUNDERS, F. J.—(1964) *Recent Prog. Horm. Res.*, **20**, 395.